# VPS35 and retromer dysfunction in Parkinson's disease

**DOI:** 10.1098/rstb.2022.0384

**Published:** 2024-04-08

**Authors:** Jordan Rowlands, Darren J. Moore

**Affiliations:** Department of Neurodegenerative Science, Van Andel Institute, Grand Rapids, MI 49503, USA

**Keywords:** Parkinson's disease (PD), *VPS35*, retromer, endosome, vesicular sorting, lysosome

## Abstract

The *vacuolar protein sorting 35 ortholog* (*VPS35*) gene encodes a core component of the retromer complex essential for the endosomal sorting and recycling of transmembrane cargo. Endo-lysosomal pathway deficits are suggested to play a role in the pathogenesis of neurodegenerative diseases, including Parkinson's disease (PD). Mutations in *VPS35* cause a late-onset, autosomal dominant form of PD, with a single missense mutation (D620N) shown to segregate with disease in PD families. Understanding how the PD-linked D620N mutation causes retromer dysfunction will provide valuable insight into the pathophysiology of PD and may advance the identification of therapeutics. D620N VPS35 can induce LRRK2 hyperactivation and impair endosomal recruitment of the WASH complex but is also linked to mitochondrial and autophagy-lysosomal pathway dysfunction and altered neurotransmitter receptor transport. The clinical similarities between *VPS35*-linked PD and sporadic PD suggest that defects observed in cellular and animal models with the *D620N VPS35* mutation may provide valuable insights into sporadic disease. In this review, we highlight the current knowledge surrounding VPS35 and its role in retromer dysfunction in PD. We provide a critical discussion of the mechanisms implicated in *VPS35*-mediated neurodegeneration in PD, as well as the interplay between VPS35 and other PD-linked gene products.

This article is part of a discussion meeting issue ‘Understanding the endo-lysosomal network in neurodegeneration’.

## 1. Introduction

Parkinson's disease (PD) is the most common movement disorder that impacts approximately 2% of the population over 65 years of age, and increases to approximately 5% in those over 85 [1,2]. Clinically, PD is characterized by cardinal motor symptoms including bradykinesia, rigidity, resting tremor and often postural instability. PD is neuropathologically characterized by the relatively selective loss of dopaminergic neurons in the nigrostriatal pathway together with a reduction in dopamine levels in the striatum, in addition to other neuronal populations [3,4]. Accompanying this neuronal loss is the formation of Lewy bodies and Lewy neurites in the brainstem that contain protein aggregates, of which α-synuclein is a major component [3,4]. Current therapies such as pharmacological replacement of dopamine and deep brain stimulation can aid in disease management and lead to sustained symptom control and quality of life for decades. To date however, there are no approved disease-modifying therapies that can slow or stop the progression of PD.

Despite PD primarily being an idiopathic disease with age being the strongest risk factor, up to 10% of PD cases are familial. These monogenic forms of PD have been linked to mutations in at least 20 distinct genes that are inherited in an autosomal dominant or recessive manner [1]. Although the exact molecular mechanisms underpinning PD pathogenesis remain unclear, the identification of these distinct disease-linked genes has aided our understanding of PD. Accordingly, an array of cellular pathways are known to be regulated by these gene products or their disease-linked mutations, including synaptic function and neurotransmission, mitochondrial turnover and activity, autophagy-lysosomal pathway and vesicular sorting pathways [5]. Notably, in monogenic forms of PD, many of the implicated gene products converge in common cellular pathways, particularly within the endo-lysosomal system [6]. One such gene of interest is *vacuolar protein sorting 35 ortholog* (*VPS35*), where an aspartic acid to asparagine mutation at residue 620 (D620N) (p.Asp620Asn, c.1858G > A) causes a late-onset, autosomal dominant form of PD that was originally identified in Swiss and Austrian families [7,8]. The VPS35 protein is a key component of the retromer complex. The retromer consists of VPS35, VPS26A or VPS26B, and VPS29, which is associated with distinct sorting nexin family members, and is essential for receiving, sorting and recycling transmembrane protein cargo from endosomes to the *trans*-Golgi network (TGN) or the plasma membrane [9]. While disruption of the retromer has been reported to result in complex cellular phenotypes, impacting degradation pathways such as mitophagy, autophagy and the lysosome, as well as the recycling of synaptic receptors [10,11], the precise nature of retromer dysfunction and its relation to neurodegeneration in PD remain unclear. Elucidating the molecular mechanisms underpinning *VPS35*-linked PD will provide important insight into the pathophysiology of familial and sporadic PD and potentially the identification of targeted therapeutics. For this to occur, however, it is critical to understand VPS35 function within the retromer complex.

## 2. VPS35 and the retromer

**Figure 1.**
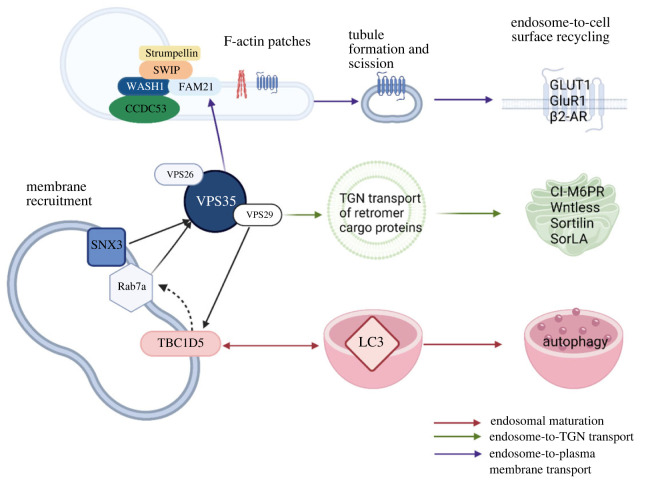
Schematic diagram of the mammalian retromer complex and its associated proteins. Depicted are the retromer-interacting proteins from studies in mammalian cells. The retromer complex consisting of VPS26, VPS29 and VPS35 is essential for recycling of endosomal transmembrane protein cargo [13]. The retromer is depicted as a single trimer here, and proteins are grouped according to function, e.g. regulators of cargo-selective retromer complex and membrane association, SNX3, Rab7a and TBC1D5. Binding directly to VPS29, TBC1D5 is a member of Tre2-Bub2-Cdc16 (TBC) family of Rab GTPase-activating proteins (GAPs); it is believed to be associated with membrane recruitment of the retromer, has been shown to bind to autophagy marker LC3 and it has been implicated to have a role in mitophagy [14–17]. The retromer facilitates two routes of cargo sorting: endosome-to-plasma membrane transport and endosome-to-*trans*-Golgi network (TGN) transport. Retromer-mediated sorting has been shown to be facilitated by association of the retromer with the pentameric WASH complex. Composed of SWIP/KIAA1033, WASH1, Strumpellin, CCDC53 and FAM21, the WASH complex is important for discrete sorting pathways by forming F-actin patches along endosomal tubes [10,18,19]. Arrows indicate relationships between respective proteins and colours indicate the cellular pathway involved, while dashed arrows indicate an interaction that has yet to be experimentally validated.

The retromer was originally identified in yeast and is highly conserved in mammals, where it acts as a master conductor to receive, dissociate and sort a diverse set of membrane cargo proteins to specialized intracellular locations [12] (figure 1). The retromer controls cellular homeostasis through the sorting and recycling of cargo to endosomal export pathways, thereby preventing their inappropriate lysosomal degradation [10,20,21]. The retromer assembles on endosomes and forms tubular vesicles that sort cargo to the plasma membrane for recycling or to the TGN via retrograde pathways (figure 1) [9,22]. Studies on the retrieval of the yeast vacuole sorting receptor VPS10p identified the necessity of the retromer for the sorting and retrieval of lysosome/vacuole hydrolase receptors, ultimately leading to the discovery of the retromer complex [12,23]. Among other known retromer cargos are various membrane proteins including transporters, ion channels, enzymes, adhesion molecules and signalling receptors.

In mammals, the retromer can be separated into two distinct associated complexes, a cargo-selective complex (CSC) trimer and a sorting nexin (SNX) dimer (figure 1) [14,19,24,25]. The CSC consists of VPS26, VPS29 and VPS35 and binds to endosomal membranes where it mediates the recognition and sorting of cargo. The SNX heterodimers consist of SNX1 or SNX2 and SNX5 or SNX6 that bind to the endosomal membrane and aid the association of the CSC with endosomal membranes through its *phox*-homology (PX) and Bin–Amphiphysin–Rvs (BAR) domains [13,21,26]. Of the CSC proteins, VPS35 is the largest subunit being composed of approximately 800 amino acids, and it is considered a highly flexible protein, forming an α-solenoid fold extending throughout the entire protein. Recently, structural analysis of the yeast retromer via cryo-electron tomography identified VPS35 homodimers forming an arch, bound by VPS26 dimers at the base (N-terminal end) and VPS29 dimers at the apex (C-terminal end). The α-solenoid fold is believed to be important in the binding of VPS29 at the C-terminal end of VPS35, whereas the N-terminus uses a PRLYL motif to bind to VPS26 [21,24,27–29]. Structural data indicate that the PD-linked D620N mutation is located adjacent to the VPS35 homodimerization interface, thereby suggesting that the mutation could reduce VPS35 dimerization efficiency and may impact the assembly and function of retromer multimers. Given this data, it is possible that the VPS35 D620N mutation also alters the binding stability between retromer and its accessory proteins, discussed below. However, the question remains as to the impact of the D620N VPS35 mutation on arch assembly. Notably, a similar structural configuration was observed in the mammalian retromer using single-particle cryo-electron microscopy and suggested that the retromer can form oligomeric species, although the function of these assemblies is not yet clear [27,30–32]. Interestingly, while VPS35 and VPS29 are largely unchanged between the mammalian and yeast retromer, VPS26 has diverged into two distinct proteins in mammals, VPS26A and VPS26B. Despite a high degree of sequence similarity, the VPS26 isoforms can bind and recycle different cargos by forming distinct retromer complexes [13,33]. Furthermore, the mammalian CSC does not form a stable interaction with the SNX dimer and requires two additional proteins, SNX3 and Rab7A, for a sustained interaction with the endosomal membrane (figure 1). Examination of mammalian cells via high-resolution microscopy revealed that the SNX dimer and CSC often exist in distinct endosomal regions, which may explain the weak interaction between these two complexes [14,34,35]. Consequently, the term 'retromer' will refer to the CSC in this review.

In the endo-lysosomal pathway, the retromer controls homeostasis through the regulation of endosomal maturation as well as via protein sorting and transport. Of note, the CSC has no membrane-binding activity and thus relies upon a variety of indirect mechanisms to associate with early and maturing endosomes. For example, in early endosomes Rab5 promotes PtdIns(3)*P* (PI3P) synthesis, which is then bound by the PX domain of SNX3, which in turn targets the CSC to early endosomes. During the early to late endosome transition, however, PI3P is converted to PtdIns(3,5)*P_2_* and Rab5 is supplanted by Rab7A, leading to endosomal maturation [34–36]. At this juncture, the retromer is responsible for recognizing and sorting distinct cargo away from lysosomes. In addition to these interactions, the retromer can associate with accessory proteins that guide cargo into discrete sorting pathways. One such interaction is with the Wiskott-Aldrich syndrome protein and SCAR homolog (WASH) complex via VPS35 (figure 1). The WASH complex is composed of WASH1, Strumpellin, FAM21, CCDC53 and SWIP/KIAA1033 [18–20]. The WASH complex interacts with VPS35 via the unstructured tail of FAM21, and its recruitment to endosomes drives the formation of F-actin patches on the endosomal membrane required for generating distinct domains for cargo sorting to both the TGN and plasma membrane [18,19,26]. In addition to interacting with the retromer, the C-terminal domain of FAM21 plays a part in a number of other protein-protein interactions including, but not limited to, the COMMD/CCDC22/CCDC93 (CCC) complex, RME-8 and FKBP15 (reviewed in [18,37–39]). Canonically, endosome-to-TGN retromer cargo includes the cation-independent mannose-6-phosphate receptor (CI-M6PR), sortilin, Wntless, and SorLA, whereas endosome-to-plasma membrane cargo includes the glucose transporter GLUT1, AMPA glutamate receptors (GluR1 and GluR2), insulin-like growth factor 1 receptor (IGF1R), and the *β*2 adrenergic receptor (*β*2-AR) (figure 1) [19,26,40]. The WASH complex is primarily involved in the sorting of GLUT1 and *β*2-AR to the plasma membrane, which is dependent on its association with SNX27 [20,26,34,35]. Additionally, the recruitment and coordination of the WASH complex with the retromer can promote tubule scission and retrieval of CI-M6PR in endosome-to-TGN transport. Interestingly, an impairment of VPS35–FAM21 binding and WASH complex recruitment to endosomes is caused by the PD-linked D620N mutation, and this molecular defect could potentially play a role in PD pathophysiology [19,20,26,41]. A recent study has shown that inhibition of TBC1D5, a GTPase-activating protein for Rab7a, can lead to the activation of Rab7a that is sufficient to rescue impaired retromer activity due to the D620N mutation by enhancing retromer recruitment to endosomes [25]. It should be noted, however, that both the WASH complex and TBC1D5 are not conserved in yeast, and thus are dispensable given that yeast are still able to operate an efficient endosome-to-Golgi retrieval pathway [14].

Coupling the ubiquitous expression of the retromer with its role in various cellular processes, such as glucose uptake and metabolism to maintaining lysosomal hydrolases, it is evident that the retromer is integral to cellular health and functioning [42]. Notably, VPS35 mRNA is detected in the mammalian brain where it is highly expressed in frontal cortex, hippocampus, striatum and cerebellum, and shows highest expression in neurons and oligodendrocytes [43]. Moreover, VPS35 is required for various cellular mechanisms, as evidenced by defects in WASH complex binding, AMPA receptor sorting, the autophagy–lysosomal pathway, and mitochondrial dynamics and activity induced by the PD-linked D620N mutation [44–47]. Interestingly, while the D620N mutation does not disrupt the interaction of VPS35 with other subunits of the retromer complex, there is some discordance within the literature surrounding the mechanism of VPS35-dependent neurodegeneration. For example, overexpression of wild-type (WT) VPS35 has been reported to protect dopaminergic neurons exposed to mitochondrial toxins *in vitro* [48], whereas a contrasting report indicates the overexpression of WT or D620N VPS35 to be neurotoxic *in vitro* and *in vivo* [49]. Extending these findings, Tsika *et al*. [49] also reported that, compared to WT VPS35, the D620N mutation had pathogenic effects leading to enhanced nigrostriatal pathway degeneration in the adult rat brain. Taken together, these reports highlight an important role for VPS35 and the retromer for normal cellular functioning throughout the body and particularly within the brain. However, our understanding of the molecular and cellular mechanisms involved in VPS35-dependent neurodegeneration in PD is still rather limited.

## 3. *VPS35* mutations in PD

The D620N mutation in *VPS35*, initially identified in a large Swiss PD family in 2011 through exome sequencing, has enabled the identification of *VPS35*-PD families from Tunisia, Israel and the United States, as well as three Austrian families [7,8,50]. Since its initial discovery, the D620N mutation has subsequently been identified in several PD families and individuals worldwide. Interestingly, the D602N mutation is rare in Asian populations, with the exception of Japanese populations, and it has been predominantly identified in individuals of Caucasian decent [51]. Although several additional rare variants in *VPS35* have been reported in individual PD subjects (i.e. R32S, P316S, R524W, I560T, H599R and M607V), only the D620N mutation has been confirmed as pathogenic owing to its segregation with disease in PD families [11].

*VPS35*-PD is clinically similar to sporadic PD with a typical late-onset of disease accompanied by cardinal motor symptoms, responsiveness to levodopa therapy and, in some subjects, mild cognitive impairment [50,52]. Due to the indistinguishable clinical observations between *VPS35*-PD and sporadic PD subjects, it would be beneficial to fully evaluate the neuropathology of *VPS35*-PD *post-mortem* cases, to understand whether similar mechanisms may underlie familial and sporadic PD. To date, however, the neuropathology of D620N *VPS35*-PD cases is unknown, as only one subject has been assessed including the cortex and parts of the basal ganglia (but critically lacking the brainstem) with no signs of Lewy pathology or α-synuclein aggregation [50]. Despite numerous studies demonstrating the importance of VPS35 and the retromer for normal cellular function and viability, the mechanisms underpinning neurodegeneration in PD induced by the D620N *VPS35* mutation remains unclear. The D620N mutation has been reported to disrupt the role of VPS35 in at least three cellular pathways, including autophagy, neurotransmission and mitochondrial dynamics/function.

## 4. Autophagy

Autophagy is a highly conserved intracellular lysosome-mediated degradative pathway that plays an important role in cellular homeostasis by sequestering various intracellular components for lysosomal delivery and degradation. In mammals, there are three primary types of autophagy: chaperone-mediated autophagy (CMA), microautophagy and macroautophagy. Macroautophagy participates in protein aggregate and organelle degradation, and can be further divided into selective autophagy classified by the type of cargo sequestered (mitophagy and aggrephagy) and non-selective macroautophagy [53–55]. Given that impaired mitochondrial turnover and function, as well as increased protein aggregation, are pathological features of PD, it is unsurprising that disruptions in autophagic pathways have been implicated as a major contributing factor to neurodegenerative diseases. In PD, several mechanisms have been proposed to perturb autophagy and lysosomal pathways, promoting the progression of PD pathology in vulnerable brain regions [4,10,55]. Notably, the retromer, in association with its accessory proteins such as the WASH complex, has been observed to have a crucial role in endosomal protein sorting and the autophagic process [56–58].

The WASH complex plays an integral role in endosome sorting and has previously been shown to be necessary for negatively regulating autophagosome formation, with WASH complex deficiency resulting in embryonic lethality and excessive autophagy [41,58]. Extending these initial findings, the D620N mutation was shown to impair retromer–WASH complex binding and endosomal recruitment through reduced affinity of VPS35 for FAM21. Additionally, the D620N mutation was observed to alter GLUT1 localization from the cell surface to an intracellular location [58]. The aberrant autophagy resulting from the impaired WASH complex binding to D620N VPS35 was attributed to the abnormal sorting of autophagy receptor ATG9A and reduced autophagosome formation. The multipass transmembrane protein ATG9A is important for autophagosome formation that localizes to the TGN and recycling endosomes under basal conditions, but it undergoes redistribution in the early stages of autophagy to compartments that become positive for the autophagosome marker LC3 [58,59]. Perturbed ATG9A sorting has been reported to result in reduced colocalization with LC3-positive autophagic structures, leading to impaired autophagosome formation [58,60–63]. Interestingly, mislocalization of ATG9A has also been observed in an α-synuclein overexpression model, often used to recapitulate familial forms of PD caused by *SNCA* (α-synuclein) gene multiplication [64]. It is important to note that while the impact of the D620N mutation on WASH complex binding in mammalian cells has since been recapitulated by other groups, it has yet to be demonstrated in brain cells specifically or directly linked to neurodegenerative processes. As such, it remains unclear whether impaired WASH complex binding to the retromer is sufficient to drive PD pathology.

Another well-studied retromer cargo, CI-M6PR, is a critical transporter of acid hydrolases—such as the aspartyl protease cathepsin D—from the TGN to the pre-lysosomal compartment. Newly synthesized lysosomal-bound enzymes arriving in the Golgi, such as pro-cathepsin D, are specifically modified with mannose-6-phosphate (M6P) groups for recognition by the CI-M6PR in the TGN and transported to the pre-lysosomal compartment [10,65]. The proper translocation of pro-cathepsin D into the late endosomal compartment is important for its maturation and proper lysosomal function. Supporting this notion, disrupted cathepsin D sorting, and consequently lysosomal dysfunction, have been demonstrated in cell lines and PD patient-derived fibroblasts expressing D620N VPS35, despite CI-M6PR sorting appearing largely normal [66,67]. Cathepsin D is considered the main lysosomal endopeptidase for the degradation of long-lived proteins, including α-synuclein. As such, it has been suggested that the disrupted lysosomal delivery of cathepsin D could therefore impair the degradation of α-synuclein, as well as other aggregation-prone proteins [67–69]. Supporting this notion, an increase in α-synuclein was observed in substantia nigra dopaminergic neurons of mice overexpressing D620N VPS35; however, cathepsin D sorting was not evaluated [68]. Extending these findings, the authors observed that sorting and degradation of LAMP2a, a key CMA protein required for α-synuclein degradation, is regulated by VPS35 [55]. Furthermore, it was seen that VPS35 deficiency decreased LAMP2a endosome-to-TGN retrieval, accelerated its degradation, enlarged LAMP1-postive lysosomal vesicles and increased α-synuclein accumulation. Similarly, LAMP2a sorting is impaired and its degradation accelerated, and α-synuclein accumulates, following the overexpression of D620N VPS35 [68].

Both the retromer and VPS35 have also been linked to the regulation of the divalent metal transporter 1 (DMT1), a transporter of Fe(II) ions, via the TGN. Disruption to iron metabolism has been reported in a variety of neurodegenerative diseases, including PD, wherein iron accumulation is observed in the dopaminergic neurons of the substantia nigra [70,71]. While the exact mechanisms are unknown, alterations in proteins critical for iron uptake have been observed, including increased expression of DMT1 and decreased iron exporter FPN1 [71,72]. Consistent with these findings, impairments to either VPS35 expression or function have been reported to missort DMT1 to the lysosome, promoting the accumulation of Fe(II) in lysosomes in SH-SY5Y neural cells [73,74]. Although the total iron content of cells was not altered by VPS35 dysfunction, *VPS35* knockdown was observed to result in a significant accumulation of α-synuclein [74]. Previous studies have demonstrated that metal ions, including copper and iron, can accelerate α-synuclein aggregation, which has been reported to perturb lysosomal degradation [75–77].

## 5. Neurotransmission defects

The efficient recycling and turnover of cellular components are essential to neuronal function and survival, enabling them to maintain functional plasticity. The retromer is localized throughout the neuron, including within the cell soma, axon and dendrites and is important for neuronal functioning, underlying delivery of various receptors to dendrites and both extrasynaptic and synaptic sites [10,44]. Consequently, the role of VPS35 and the retromer in this area is of great interest in neurodegenerative diseases. Normally, VPS35 and the retromer have been seen to localize to dendritic spines, wherein they facilitate binding and sorting of the AMPA receptor GluR1 [46].

Overexpression of WT VPS35 in primary rodent neurons has been shown to impact synapse number and AMPA receptor localization [46]. Overexpression of D620N VPS35 however, was shown to alter VPS35 localization and motility at the dendrite, disrupt GluR1 sorting and increase colocalization of VPS35 with GluR1, indicating a potential dysfunction in glutamatergic signalling [46]. Additionally, human induced pluripotent stem cell (iPSC)-derived dopaminergic neurons harbouring the D620N mutation were also observed to have an increase in GluR1 cluster intensity. Supporting these findings, a recent study by Kadgein *et al.* [45] observed GluR1 association with VPS35 in neurons derived from a *D620N VPS35* knockin mouse model. While previous studies have used overexpression of VPS35 variants, *D620N VPS35* knockin mice provide a more physiologically relevant model, expressing VPS35 at endogenous levels. *D620N VPS35* knockin mice models were also observed to have increased glutamatergic activity in primary neuronal cultures, which was associated with increased GluR1 surface expression and glutamate transmission [45]. By contrast, *VPS35* knockout mice have perturbed neurotransmission. In this model, depletion of *VPS35* decreased surface expression of GluR1 and GluR2, accompanied by impaired dendritic spine maturation, which is suggested to be a consequence of abnormal GluR2 sorting [78].

Furthermore, *D620N VPS35* knockin mice have been observed to have increased dopamine release, elevated dopamine metabolites and a significant reduction in dopamine transporter (DAT) levels in the striatum in mice as young as 3 months old [79]. Other studies using independent knockin models, however, have reported contrary findings, with one reporting a decrease in striatal dopamine [80], another an increase in striatal dopamine but not dopamine metabolites [81], and an additional study that observed no change in extracellular striatal dopamine even at 15 months [56]. While genetic background or age could have a significant contribution to the discrepancies between these *D620N VPS35* knockin mice, as well as the methodology used for detecting dopamine, it is evident that further investigations are required.

## 6. Impaired mitochondrial dynamics and function

Perturbed mitochondrial homeostasis has been consistently implicated in the pathogenesis of familial and sporadic PD. Impairments in several mitochondrial processes have been reported in a range of PD models including defects in mitophagy, dynamics, biogenesis, defective calcium homeostasis and mitochondrial DNA mutations [10,11,40]. The retromer complex has previously been implicated in mediating vesicle transport from mitochondria to lysosomes and peroxisomes by facilitating the formation of mitochondrial-derived vesicles (MDVs). MDV formation and delivery ultimately enables the removal and recycling of mitochondrial contents [82]. MDV formation and delivery were shown to be reliant upon interactions of VPS35 and VPS26A with the mitochondrial-anchored protein ligase (MAPL). MAPL is a mitochondrial small ubiquitin-like modifier (SUMO) E3 ligase that is reported to positively regulate the mitochondrial fission GTPase Drp1 [82,83]. VPS35 and the retromer have also been suggested to have a broader role in mitochondrial maintenance, wherein they may regulate mitochondrial fission and fusion events potentially directly or indirectly, yet endogenous VPS35 has not been convincingly demonstrated to exhibit localization to mitochondria in different cell types.

Recently, VPS35 has been observed to interact with Drp1 and regulate mitochondrial fission. Using rodent primary neurons it was observed that overexpression of human VPS35 led to mitochondrial fragmentation and neuronal cell death. These phenotypes were further enhanced in the presence of the D620N mutation relative to WT or R524W VPS35 [84]. Moreover, these effects were seen in fibroblasts derived from PD subjects harbouring the D620N mutation, as well as in *VPS35* knockdown and rescue experiments in M17 neural cells, indicating that the effects of D620N VPS35 are independent of potential overexpression artefacts [84]. Increased mitochondrial fragmentation has also been observed following WT or D620N VPS35 overexpression in mouse substantia nigra neurons *in vivo* [84], as well as in the substantia nigra of heterozygous *D620N VPS35* knockin mice at 16 months of age [85]. Accompanying this mitochondrial fragmentation, *VPS35* mutations were observed to impair mitochondrial respiration, decrease membrane potential, reduce ATP levels and increase reactive oxygen species production [84]. Notably, use of mdivi-1, a selective inhibitor of dynamin-related GTPases including Drp1, was able to rescue the observed mitochondrial fragmentation in mice expressing D620N VPS35 and patient-derived D620N PD fibroblasts, as well as restore the defects in mitochondrial respiration [84]. While further investigation is still required, these studies highlight a potential role for altered mitochondrial dynamics in D620N VPS35-induced neurotoxicity, although it remains unclear whether these effects occur directly or result indirectly as a consequence of altered autophagy (mitophagy) and lysosomal activity.

Extending these findings, ablation of *VPS35*, both *in vitro* and *in vivo*, has been observed to increase mitochondrial fragmentation and lead to abnormal mitochondrial morphology [47]. These *VPS35* knockout neurons were reported to have an increase in the levels of the mitochondrial E3 ubiquitin protein ligase 1 (MUL1) (MAPL) and a resultant decrease in the mitochondrial fusion protein, mitofusin 2 (Mfn2). These mitochondrial dynamic changes were accompanied by altered mitochondrial respiration and function [47]. Increased MUL1 levels and decreased Mfn2 were also observed following the overexpression of PD-linked VPS35 mutants. Notably, the mitochondrial defects in *VPS35* knockout neurons were rescued by exogenous expression of WT VPS35, but not the D620N mutant [47]. Although this study suggests that *VPS35* mutations act through a loss-of-function mechanism in the context of mitochondrial dynamics, several of the known functions of the retromer are not compromised by these PD-linked mutations [10,41,46,49,58]. Furthermore, while the loss of nigral dopaminergic neurons in conditional *VPS35* knockout mice recapitulates PD-like pathology, the relevance of this model to *VPS35*-linked PD is questionable due to the subtle effects of the D620N mutation compared to global retromer impairment in *VPS35* knockout mice. This is perhaps best exemplified by the observation that germline homozygous *VPS35* knockout mice are early embryonic lethal whereas *D620N VPS35* knockin mice are viable and exhibit a normal lifespan [56,86].

## 7. *VPS35* and other PD-linked genes

Given the diverse interactions of VPS35 and the retromer, it is unsurprising that they have been linked to the products of other PD genes, including *LRRK2, parkin* and *α-synuclein*, which may converge on common pathways to augment neurodegeneration in PD [11]. Previous studies have identified a genetic interaction between *VPS35* and *parkin* [87], including a link to MDV formation [82,88]. Studies in *Drosophila* models posited that due to their shared role in MDV formation, an interaction may exist between VPS35 and parkin, and/or PINK1. While it was established that *VPS35* and *parkin* have a genetic interaction, with VPS35 likely downstream of parkin, how parkin contributes to the pathogenicity of *VPS35* mutations in PD remains unclear [87,88]. In this regard, it has been shown that VPS35 can serve as a substrate for the E3 ubiquitin ligase activity of parkin, which modifies VPS35 with non-degradative poly-ubiquitin chains [89]. The knockdown of *parkin* in primary neurons led to the modulation of WASH complex components and abnormal sorting of ATG9A [89], a retromer cargo. Consistent with *Drosophila* studies, parkin expression was not required for dopaminergic neuronal degeneration induced by human D620N VPS35 expression in mice [89], instead supporting a role for parkin upstream of VPS35.

As mentioned, *VPS35* deficiency has been reported to increase α-synuclein accumulation via impaired lysosomal function [46,68,76]. *VPS35* depletion can impair the lysosomal degradation of α-synuclein through the abnormal sorting of cathepsin D and DMT1, while overexpression of the D620N VPS35 mutant impairs CMA of α-synuclein by disrupting LAMP2a sorting [66,68,73,74]. Additionally, *VPS35* loss-of-function has been implicated in increasing the toxicity induced by human α-synuclein variants in yeast and worm models [90]. In addition, hippocampal neuronal loss observed in transgenic mice expressing human WT α-synuclein was rescued by the overexpression of WT VPS35, but not by the PD-linked P316S variant, or by *VPS35* silencing [90]. By contrast, a recent study demonstrated that overexpression of WT VPS35 failed to protect against nigral dopaminergic neurodegeneration induced by the viral-mediated expression of human WT α-synuclein in a rat model of PD [91]. Furthermore, it was shown that endogenous α-synuclein was not required for nigrostriatal pathway dopaminergic neurodegeneration induced by the viral-mediated expression of human D620N VPS35 in mice. In contrast to reports from simpler models, the authors also reported that the lethal neurodegenerative phenotype exhibited by human A53T-α-synuclein transgenic mice was not altered by the germline heterozygous deletion of *VPS35*, and that A53T-α-synuclein transgenic mice did not exhibit evidence for a retromer deficiency in vulnerable brain regions of symptomatic mice [91]. Collectively, these data suggest a rather limited interaction between α-synuclein and VPS35 in neurodegenerative models of PD, but it remains possible that both proteins may indirectly converge on a common pathophysiological pathway such as lysosomal degradation.

VPS35 and LRRK2 have previously been reported to interact with each other through co-immunoprecipitation assays in SH-SY5Y cells [92]. It is also suggested that these two proteins are involved in a similar pathogenic mechanism because the CI-M6PR sorting defect induced by D620N VPS35 is phenocopied by G2019S LRRK2 overexpression and can be rescued by WT but not D620N VPS35. In addition, overexpression of WT VPS35 has been demonstrated to rescue retinal degeneration in a *Drosophila* model of PD induced by PD-linked LRRK2 mutants [93]. These studies support a mechanism whereby PD-linked *LRRK2* mutations may induce a downstream retromer deficiency. More recently, PD-linked *VPS35* mutations have been reported to lie upstream of LRRK2 as the D620N mutation can induce robust LRRK2 kinase hyperactivation. Specifically, this was observed as a 2–6-fold increase in the LRRK2-mediated phosphorylation of its substrates Rab8a, Rab10 and Rab12 in mouse embryonic fibroblasts (MEFs) and multiple tissues from *D620N VPS35* knockin mice. Increased Rab phosphorylation was shown to be dependent on LRRK2 kinase activity, as it was reversed in *D620N* knockin MEFs and tissues by treatment with the selective LRRK2 kinase inhibitor, MLi-2 [94]. Notably, heterozygous *D620N* PD patient-derived neutrophils and monocytes also exhibit increased LRRK2-dependent Rab10 phosphorylation [94]. As the retromer does not possess catalytic activity, the increased LRRK2 substrate phosphorylation induced by the *D620N* mutation may indicate an altered subcellular localization of LRRK2 and its Rabs leading to increased substrate access, and/or the recruitment of an unknown LRRK2 regulator via an unclear mechanism.

## 8. Conclusion and future perspectives

While the molecular mechanisms underlying neurodegeneration induced by the PD-linked *D620N VPS35* mutation have yet to be fully elucidated, they have firmly established a role for endosomal retromer sorting in the pathogenesis of PD. With only a single missense mutation so far confirmed to be pathogenic (i.e. D620N), numerous experimental models have been developed to understand the mechanisms involved in *VPS35*-linked PD. The use of *VPS35* knockout or overexpression in rodents has proven useful, but it can complicate the identification of specific disease mechanisms as both approaches can often lead to severe phenotypes and susceptibilities unrelated to PD [43,95,96]. Accordingly, the development of *VPS35* knockin mouse models has suggested that the D620N mutation may have rather subtle and/or selective effects on overall retromer function and neuronal damage, when compared to knockout models. Notably however, most rodent models of PD-linked mutations based on knockout or knockin approaches have largely failed to reproduce key aspects of human PD neuropathology, and thus findings from these *VPS35* models should still be taken with some caution.

Retromer dysfunction in PD may culminate in abnormal mitochondrial turnover and activity, neurotransmission and/or macroautophagy. The use of human iPSC-derived brain cell models has also contributed to our understanding of PD pathobiology, and for the *D620N VPS35* mutation these models have initially suggested altered autophagy-lysosomal pathway activity, α-synuclein accumulation, abnormal AMPA receptor sorting and mitochondrial dysfunction in neurons [46,97,98]. While iPSCs can be used to study human brain cells, there are several limitations to consider. One such limitation is the association of PD and ageing, which consequently requires the development of an aged iPSC model to recapitulate hallmark phenotypes of PD such as dopaminergic neuronal degeneration [99]. Although several studies have used factors to accelerate ageing and better represent the disease, such as progerin or telomerase inhibitor 2-[(E)-3-naphthalen-2-yl-but-2-enoylamino]-benzoic acid (BIBR1532), these factors may not capture all aspects of normal ageing [100,101]. Further limiting iPSC modelling is the lack of neuronal complexity as compared to studies in the rodent brain. This is partially due to the two-dimensional monolayer system of iPSCs coupled with the efficiency of different differentiation protocols [99]. Regardless of these limitations, the use of PD patient-derived iPSCs will likely provide novel insights into disease progression and gene-specific mechanisms of PD pathogenesis, including for *VPS35*.

Emerging from studies of VPS35 so far, is the notion that the PD-linked D620N VPS35 protein appears to be largely functional, and does not confer a complete loss-of-function effect unlike *VPS35* depletion models [10,56,66,87,91]. However, whether the *D620N VPS35* mutation acts via a toxic gain-of-function or partial loss-of-function mechanism remains unclear. For example, D620N VPS35 can promote LRRK2 hyperactivation yet also exhibits an impaired interaction and endosomal recruitment of the WASH complex [25,94], but neither phenotype has been shown to drive neuronal damage. As such, it is not yet clear how best to selectively target the retromer in PD for therapeutic development, but this could potentially involve LRRK2 kinase inhibition [94], promoting the WASH complex interaction (via TBC1D5 inhibition) [25], or boosting retromer stability via pharmacological chaperones [102]. Although the exact pathogenic mechanisms underlying D620N VPS35-mediated neurodegeneration are not yet clear, several cellular pathways downstream of retromer dysfunction have been implicated, including mitochondrial fusion and fission [47,84], autophagy defects [58,67,68], altered neuronal signalling/transmission [45,46,78] and interactions with other PD-linked gene products [10,64,85,87,92,93]. Future studies elucidating such mechanisms will be beneficial in the development of therapeutics for *VPS35*-linked PD and potentially sporadic PD. Retromer dysfunction has also been implicated in other neurodegenerative diseases (i.e. Alzheimer's disease, tauopathies, amyotrophic lateral sclerosis) [10], so insights from studying PD-specific mechanisms may also contribute to our understanding and therapeutic development for these devastating diseases.

## Data Availability

This article has no additional data.
